# Herping the African Continent: Alien Amphibians and Reptiles in Sub-Saharan Africa

**DOI:** 10.3390/biology15080639

**Published:** 2026-04-18

**Authors:** Grzegorz Kopij

**Affiliations:** Department of Animal Ecology & Biology, Wrocław University of Environmental & Life Sciences, ul. Kozuchowska 5b, 51-631 Wroclaw, Poland; grzegorz.kopij@upwr.edu.pl

**Keywords:** nature conservation, wildlife management, invasive species, introductions, island biogeography

## Abstract

Introduction of species represents today one of the most important problems of nature conservation. Special attention is paid to alien vascular plants and vertebrates. In the Afrotropical Region (sub-Saharan Africa), however, there is a lack of comprehensive review of alien amphibians and reptiles. The presented paper constitutes an attempt to overview the status, distribution and threats posed by introduced herp species to sub-Saharan Africa since the second half of the 18th century. This review includes 21 amphibian (including 10 established) and 57 reptile (including 33 established) species introduced to sub-Saharan Africa. Most species introduced to sub-Saharan Africa which subsequently developed viable populations originated from the Malagasy (32%), Afrotropical (30%), and Oriental (27%) Regions. Most introductions were made in the last two decades, mostly as results of an increase in international trade and herp pet industry, especially in South Africa. Stowaway and pet trade are the most common pathways of introductions. Several factors determine the successful establishment of introduced alien herp species in sub-Saharan Africa, viz. behavioral and morphological traits, propagule pressure, climate and habitat overlap, and presence of potentially competing species. The impact of alien herps in sub-Saharan Africa on the local biodiversity is not well investigated. In comparison with other continents the number of introduced and established herp species in sub-Saharan Africa is relatively low. The Malagasy Region has the highest number of introduced herp species in sub-Saharan Africa.

## Simple Summary

Introductions of alien species may cause enormous ecological problems. It is, therefore, important to closely monitor their introduction and subsequent spread. The presented paper comprises an overview of alien amphibians and reptiles in sub-Saharan Africa. The current status of these alien species is assessed. Their impact on the indigenous species is relatively low in the mainland, but high in the Mascarenes and other small islands surrounding Madagascar. However, recently their introduction rate has greatly accelerated everywhere mainly due to increased pet trade, so it is important to keep all records regarding their occurrence to prevent further spread.

## 1. Introduction

In recent years, one of the most important problems in nature conservation is the introduction of alien species, especially those that subsequently become invasive and expand. Their negative impacts on local biodiversity are well documented throughout the world. These include competitive exclusion, dislocation, increased predation rate, spread of alien parasites and diseases, hybridization and others [1]. Also, human health and the economy can be negatively affected by invasive species through the spread of zoonotic diseases (e.g., aspergillosis or Newcastle disease) and transmission of pathogenic organisms (e.g., malaria, salmonellosis), e.g., [2,3,4,5,6]. Preventing the introduction or invasion is the best management tool. Combating and eradicating invasive species often pose enormous difficulties to local nature conservation and government authorities. Early detection of such introduction or invasion stage is often an effective tool in the fight if the response is rapid. Species with high risk that are well established, or medium risk with high potential for expansion, should be a management priority to prevent further expansion into new areas within the region. However, early warning and adequate management are based on the knowledge derived from regular monitoring programs [7].

With regard to introduced animals, vertebrates attracted the highest attention of researchers, due to their larger size and the effect on local biodiversity. Therefore, the group is also more intensively studied than other animals. Such studies are, however, not equally intensively conducted in the world. While in Europe [8] and North America [9], the introduced vertebrates are closely monitored, in most tropical countries they often pass unnoticed. This remains true especially in regard to small species, such as frogs and lizards. In recent decades, amphibians and reptiles have been popular as pets; they can easily escape from terrariums into the wild, and may subsequently survive, reproduce, develop viable populations, expand, and become invasive species.

In sub-Saharan Africa, introduced birds and mammals have been subjects of recent reviews [10,11], whereas the introduction of alien amphibians and reptiles has been closely monitored only in South Africa [12,13], Madagascar, Seychelles and the Mascarenes [14,15,16,17]. In most other countries, this issue remains neglected. Moreover, the invasion of alien amphibians and reptiles has significantly accelerated at the end of the 20th century [18,19] and there is an urgent need for an overview of the status of these animals in the whole Afrotropical Region. This is the purpose of the presented work. Specifically, it (1) reports on all known cases of introduced amphibians and reptiles; (2) assesses their current invasive status; (3) evaluates their impact on the local biodiversity; and (4) outlines directions for management of the alien species in all African countries south of the Sahara.

## 2. Materials and Methods

All amphibian and reptile species ever introduced to Africa and its islands are the subject of this review. Detailed accounts are provided for species that have developed viable populations.

The literature on amphibian and reptile introductions in Africa south of the Sahara was reviewed from 1950 to present. Islands geographically belonging to Africa were also included in this review, i.e., Cape Verde, São Tome and Principe, Bioko and Annobón of Equatorial Guinea, Madagascar with small oceanic islands around such as the Mascarenes (Mauritius, Reunion, Rodrigues and others), Seychelles, Comoros, Mafia, Zanzibar, Pemba off Tanzania, and Socotra in the Gulf of Aden.

To search the relevant literature, the Google Scholar bibliographic database (URL: scholar.google.com; accessed on 16 January 2026) was used. The following keywords were applied: introduced amphibian/reptile species (or alien amphibian/reptile species) + Africa (or particular African country or specific island), e.g., *Lepidodactylus lugubris* + Mauritius. The particular amphibian/reptile species (both common and scientific names) were also used as a keyword + Africa (or the particular African country or specific island), e.g., *Emys orbicularis* + Africa, common slider + Namibia, etc. To investigate the impact of alien amphibians and reptiles on local fauna, the following keywords were applied: extinct (or threatened or endangered) + mammals (or birds, reptiles, amphibians, or fishes) + Africa (or the particular African country or the particular African island), e.g., extinct + amphibians + Africa. Special websites dealing with introduced amphibian/reptile species were also consulted through direct searches on the internet.

The nomenclature and systematics of reptiles follow [20], while amphibians follow [21]. The conservation status of each species was assessed according to the IUCN Red List of Threatened Species [22].

Four categories/levels of introduction are used in this work:

1. Introduced, but has not developed a viable population;
2. Introduced and has developed a viable population, but the population has very restricted range, is unstable and is not expanding;
3. Introduced and developed a viable population, but subsequently declined and finally became extinct;
4. Introduced, developed viable population and is stable or expanding and may be invasive.

Main features of an invasive alien taxon are establishment of self-sustaining populations and their significant effect on native ecosystems. Species which occupy their original range are called native or indigenous. Those which were brought by humans to other places are alien or introduced. Three types of introduction/invasion are distinguished according to the geographical location/distance: transoceanic, continental and local (translocation). If introduction is initiated by escape from terrariums, it is usually unintentional. In this case, the dates of release are not well known. Introduction can be intentional or unintentional. Introduced species become invasive if they are spreading rapidly and negatively affecting indigenous species. If an introduced species does not spread and establishes only a small local population, not affecting native species, it is regarded as not invasive. An established species reproduces and establishes a viable population. Depending on when a species was introduced, the following types of introduction can be distinguished: ancient (before 1800), historical (after 1800) and recent (last 25 years).

The following terms are used to determine the way of introduction of a particular species to a new area/range, based on [23].

1. Unintentional translocation: Transportation of habitat or nursery materials, as accidentally transported contaminants of the horticultural trade, within consignments of wood, in construction materials, e.g., by stowaway, airplanes, or camping vehicles.
2. Intentional translocation, e.g., by intentional release from a terrarium.
3. Relocation: Done within the species range.
4. Repatriation: Natural return to the abandoned areas of former occurrence.
5. Reintroduction: Return to the natural range with the help of humans.

After careful consideration, if a species’ occurrence was proven as a result of repatriation or reintroduction, it was excluded from the presented review, due to not actually being an introduced or alien species.

## 3. The Introduced Species

All species introduced to sub-Saharan Africa (including the Malagasy Region) are listed in Table A1 and their familiar affinities in Table 1.

### 3.1. Species Which Have Developed Viable Populations and Are Expanding/Invasive

Invasiveness has been suspected for many introduced species, but has not been evidenced or has been poorly investigated. All these species were not treated as invasive in the presented review. Also, the fact that some species are invasive in other parts of the world does not necessarily mean that they are also invasive in Africa. Most of the evidence of invasiveness (with relevant references) is listed in Chapter 9 (Impacts of Introduced Amphibians and Reptiles on Local Fauna).

#### 3.1.1. African Clawed Frog *Xenopus laevis*

The African clawed frog is endemic to South Africa, but invasive in North America, South America, Asia and Europe [24]. African clawed frogs were formerly bred for pregnancy testing of people, and at present are kept as laboratory organisms and as pets [13]. In South Africa, the species has expanded its range by occupying artificial impoundments, and is used as a bait by fishermen [13]. As these animals appear to be commercially available, it is likely that small numbers are moved by fishermen. Additionally, fishermen are known to seed dams with the African clawed frog in order to produce a local supply of live bait. Each of these practices is likely to propel propagules of the African clawed frog into new water bodies through jump dispersal. This is not to say that these animals cannot reach isolated water bodies through their own diffusion-based dispersal [13].

#### 3.1.2. Guttural Toad *Sclerophrys gutturalis*

The only southern African invasive population of the guttural toad is known from a peri-urban area in Cape Town (Constantia since 2000, and Bishopscourt since 2015). The spread of this toad is thought to be mainly through a leading-edge dispersal, but two confirmed instances of jump dispersal are also known. An eradication scheme was passed to remove the species. From 2010 to 2015, more than 5000 post-metamorphic toads and thousands of eggs and tadpoles were removed. Despite this, the species is still expanding around Cape Town. The guttural toad was intentionally introduced (probably from the Durban area) to Mauritius and from there to Reunion in the 1920s as a biological control for mosquitoes [25].

#### 3.1.3. Cane Toad *Rhinella marina*

The cane toad is a Neotropical species. It was introduced to Mauritius from South Africa in 1922 to control sugarcane cane beetles. Subsequently, it has spread across the island, thriving and adapting by shrinking in body size by up to a third. It is considered a threat to native fauna due to its diet [2,26,27]. Introduced to many other parts of the world, i.e., Hawaii, the Caribbean, Florida (USA), Australia and Oceania. It has become a pest in many host countries, and poses a serious threat to native animals [26,27].

#### 3.1.4. Common Slider *Trachemys scripta*

The common slider was introduced to South Africa, Kenya, Seychelles Mauritius and Reunion. It is rather scarce and restricted, but probably expanding in Africa, including South Africa. Globally it is very widespread, as an expanding and invasive alien species [28]. It is widespread and common especially in Europe and SE Asia (reported in 68 Eurasian countries), and has also been recorded in Australia, New Zealand, the Middle East, the Canary Islands, the Azores, Hawaii, and several other Pacific islands [28,29].

#### 3.1.5. Brahminy Blind Snake *Indotyphlops braminus*

The Brahminy blind snake or flowerpot snake originates from Southeast Asia, but has become invasive all over the world and is, after the common slider, the world’s most widely distributed reptile [18]. This was one of the first introduced snake species recorded in South Africa (in 1838), and was only recognized as invasive in 1978. Since then, new populations have been found on the coast in Durban [30], and inland in the Western Cape. This species reproduces parthenogenetically, so it can easily establish new populations after introduction. However, the impact of these small thread snakes has not been assessed anywhere [31].

#### 3.1.6. Indian Wolf Snake *Lycodon aulicus capucinus*

The Indian wolf snake is a non-venomous snake native to SE Asia, i.e., Pakistan, India, Sri Lanka, Indochina, the Malay Peninsula, Java, the Philippines, and Timor. It is one of the most common snake species in India. In Africa, it has been introduced to Mascarenes (Reunion before 1864, and Mauritius before 1888) [32] and Cape Town. Beyond Africa, it was introduced to Hong Kong and the neighboring Guishan Island of Guangdong Province, China [33], and Christmas Island (Indian Ocean) in 1987 [34]. It was probably also introduced to other islands in the ocean, i.e., Andaman, Nicobar and the Maldives.

#### 3.1.7. Common Agama *Agama agama*

In Africa, the common agama was introduced in 1998 to the Comoro Islands, to the Cape Verde Islands in 2006 [35] and Antananarivo (Madagascar) in 2004 [36]. It was also introduced (released as a pet) to Malta in 1979 and to Florida (USA) in the 1970s, where it became a very common species [37].

#### 3.1.8. Green Tree Lizard *Calotes versicolor*

The green tree lizard or oriental garden lizard is an Indo-Malayan species (India, Sri Lanka, Nepal, Bangladesh, Pakistan, southern China, Thailand, Malaysia and Indonesia). In Africa it has been introduced to Reunion, Rodrigues Island, Seychelles [38] and recently the SW coast of Kenya. It has also been introduced to other parts of the world, i.e., France, Belgium, Florida (USA), NE Egypt, Oman, SW Australia, Brunei, Celebes, Singapore, and other islands in the Indo-Pacific [39].

#### 3.1.9. West Madagascan Clawless Gecko *Ebenavia boettgeri*

It is native to Madagascar. It has been introduced to Mauritius, the Comoro Archipelago (including the French territory of Mayotte) and Pemba Island (Tanzania) [40].

#### 3.1.10. Common Mourning Gecko *Lepidodactylus lugubris*

This species is a day gecko native to the Indo-Malayan Region but its commensal habits have led it to invading many urban areas of South Africa, such that it has been described as South Africa’s most successful invasive reptile. The earliest records date back to around 1956 in Port Elizabeth, although other introductions may have been earlier [41]. Expansions in peri-urban areas of Port Elizabeth and Bloemfontein have been rapid, while that in Cape Town has been comparatively slow. As no other day geckos are native to the invaded areas, there is unlikely to be any intra-guild competition. The common dwarf gecko is not known to be invasive elsewhere in the world as its impact has not been assessed.

#### 3.1.11. Moorish Wall Gecko *Tarentola mauritanica*

It is native to the Mediterranean Region. There is an isolated introduced population in the southern Western Sahara (south of the Tropic of Cancer; therefore, it can be regarded as Afrotropical Region) as well as in Bulgaria [42], the Levant, Port-Cros Island (Hyères Archipelago, Var department, France) [43], and Rhodes (Greece) [44]. The adoption of this species as a pet has also led to populations becoming established in Florida (USA), Mexico and Argentina.

#### 3.1.12. Common Four-Toed Gecko *Gehyra mutilata*

It is an Oriental species. In Africa, it has been introduced (released as a pet) to the Mascarene Islands and Seychelles. It has also been introduced to Sri Lanka, Indochina, S California (USA), W Mexico, French Polynesia and probably many other small Indo-Pacific oceanic islands (including Hawaii).

#### 3.1.13. Day Geckos *Phelsuma* spp.

The genus *Phelsuma* is represented by over 70 species of diurnal and arboreal geckos. Most have vibrant green, blue, red, or orange coloration. They are indigenous to Madagascar and other surrounding islands [45,46]. Day geckos (and most of the other small diurnal geckos) were imported in some numbers in the 1980s and early 1990s when Madagascar allowed for the exporting large numbers of its herptile species. Luckily, there has been a dedicated group of gecko breeders who have specialized in breeding these beautiful lizards since these early times, and captive-hatched specimens are available at larger reptile shows, pet stores, and online [12].

Out of four *Phelsuma* species living in Seychelles, two are endemic (*P. sunbergi* and *P. astriata*) and the other two are introduced (*P. laticauda* and *P. abbotti*). Other *Phelsuma* species introduced in sub-Saharan Africa include the following [46,47]:

- *Phelsuma astriata* (Seychelles day gecko) from Seychelles has been introduced to Reunion.
- *Phelsuma borbonica agalegae* (Agalega day gecko) from Mauritius has been introduced to Reunion recently.
- *Phelsuma cepediana* (blue-tailed day gecko) from Mauritius has been introduced to Rodrigues.
- *Phelsuma dubia* (dull day gecko) from W and N Madagascar has been introduced in the four major Comoro islands, Zanzibar Island, Mozambique Island (Mozambique), and small coastal areas of Tanzania and Kenya.
- *Phelsuma grandis* (Madagascar giant day gecko) from N Madagascar has been introduced to Reunion and Mauritius and also to Florida and Hawaii.
- *Phelsuma laticauda laticauda* (gold dust day gecko) from Madagascar. It has been introduced to the Comoro Islands (Mayotte and Anjouan), the southern Seychelles Islands (Farquhar, Cerf, and Providence), the Mascarene Islands (Réunion and Mauritius), French Polynesia, Hawaii, and Florida.

#### 3.1.14. House Geckos *Hemidactylus* spp.

The genus *Hemidactylus* has 194 described species (Reptile Database) distributed in the tropical regions of the Old World, most in southern Asia. Seven species inhabit Indian Ocean islands; ten are from mainland Africa. *Hemidactylus mabouia* from Africa and *H. frenatus* from Asia have intercontinental distribution. The latter is the most widely distributed gecko in the world. Five invasive *Hemidactylus* species now have pantropical distributions: *H. parvimaculatus*, *H. brookii*, *H. frenatus*, *H. garnotii* and *H. turcicus* [40,48].

- *H. mabouia* sensu stricte (African house gecko). Native to sub-Saharan Africa (excluding SW, NE, most W Africa and the Malagasy Region). Introduced to various regions of the tropical world, including parts of Africa originally not occupied by the species. At present, it has pantropical distribution.
- *H. frenatus* (common house gecko). Native to Southeast Asia, spread via ships and cargo. Introduced to Mauritius before 1770 [49]. Introduced also to Comoros, and beyond Africa to Australia, the Americas, and islands globally.
- *H. mercatorius,* the former *H. mabouia* (Farquhar half-toed gecko). From Madagascar, Comoros and Aldabra. Introduced successfully to Reunion in 2010, apparently from Mayotte [46]. Introduced also to Seychelles: to Mahe in 1905, but apparently extinct, and again introduced there in 1995 and 2002. Unsuccessful multi-introductions in Platte and Frigate.
- *H. parvimaculatus* (spotted house gecko). Native to the Oriental Region. It is a part of *H. brooksi* species complex. Recognized as separate species only in 2010. Populations of *H. mabouia* species invaded West Africa, Reunion and Mauritius in the mid-1990s. Beyond Africa, it was introduced to Florida (USA) and Hawaii, the Caribbean, South America and Florida. Invasions have resulted in displacement of native geckos in Florida and Curaçao [9].
- *H. turcicus* (Turkish gecko). It is from the Mediterranean Region. Introduced to many parts of the world, with similar urban settings, e.g., East Africa, South America, the Caribbean, southern USA.
- *H. granotii* (Granot’s house gecko). Indo-Pacific parthenogenetic species. Introduced to Seychelles, New Zealand, Hawaii, Fiji, the Bahamas, Costa Rica, Guatemala, Colombia, and tropical United States (Hawaii, Florida, Georgia, Texas and California).

### 3.2. Species Which Have Developed Viable Population but Are Not Expanding/Invasive

#### 3.2.1. Frogs

The painted reed frog *Hyperolius marmoratus* from SE Africa was introduced to Villiersdorp, Western Cape (South Africa), in 1997 and to Cape Town in 2004, by using a combination of human-mediated jump dispersal and artificial impoundments [50]. The permanence of the dams mitigated the climatic barriers that prevented expansion of this species into drier and more unstable habitats [51]. In a similar way, the tinker reed frog *Hyperolius tuberilinguis*, also from SE Africa, was introduced to Cape Town and Bloemfontein.

Only one anuran species from the family Bufonidae, the Asian common toad *Duttaphrynus melanostictus,* has been introduced in 2013 (via stowaway, with a consignment of furniture) from the SE Asia Region to Tokai and Belville in the Western Cape, South Africa, and to Cape Town; and in 2011 via shipping containers to Toamasina (Madagascar), where in 2014 it occupied c. 100 km^2^. The Mascarene grass frog *Ptychadena mascareniensis* was introduced from Madagascar to Reunion as early as 1790 or before and to Seychelles in the 2010s. Three other anuran species, the African red toad *Schismaderma carens,* gray foam-nest tree frog *Chiromantis xerampelina* and African bull frog *Pyxicephalus adspersus,* were transported from SE Africa to various parts of the Western Cape. The transport was via stowaway.

#### 3.2.2. Turtles

Four highly threatened species were introduced to the Mascarenes from the Indo-Malayan Region. The wattle-necked softshell turtle *Palea steindachneri* is critically endangered. It lives in China, Vietnam and Laos. In the 1990s, it was introduced (released into the wild as a pet) to Mauritius, Reunion and Hawaii. Ephemeral releases were also recorded in N Germany. The radiated tortoise *Astrochelys radiata*, native to Madagascar, is critically endangered, and was introduced to Mauritius (Rodrigues, Round Island) and Reunion. The Northern Madagascar Spur Tortoise *Astrochelys yniphora* is the largest and rarest tortoise species in Madagascar, critically endangered (c. 450 ind.). It is endemic to dry forests in the Baly Bay area, NW Madagascar [52]. In terrariums, 80 individuals are kept in Thailand and 116 individuals in China [53,54]. The Sunda soft-shell turtle *Amyda cartilaginea* is a vulnerable Indo-Malayan species. It was introduced to Mauritius in the 1980s, where it is now well established. It was also introduced to Yunnan (China), Indonesia (Lesser Sunda Islands, Celebes, Moluccas), and S Iraq in the Persian Gulf.

#### 3.2.3. Chameleons

There are six African chameleon species which have been introduced to other parts of Africa (usually as a result of translocation), viz. the panther chameleon *Furcifer pardalis,* Cape dwarf chameleon *Bradypodion pumilum,* green giant chameleon *Calumma parsonii,* flap-necked chameleon *Chamaeleo dilepis,* and dwarf chameleon *Bradypodion* spp. They are, however, not expanding (Mascarenes), and some have even become extinct (South Africa). From 1977 to 2001, 66 importing countries and 70 exporting countries were recorded as being involved in the chameleon trade. In Africa, these countries include mostly Madagascar, Tanzania, Kenya and Togo [55].

#### 3.2.4. Skinks

Although the family Scincidae is the most speciose reptile family, only one species, the Bouton’s snake-eyed skink *Cryptoblepharus boutoni* has been introduced from Mauritius, where it is an endemic species, to mainland sub-Saharan Africa (Table 1). It occurs in scattered island or coastal populations in the Indian Ocean. The most southern known population exists on Black Rock on the northern coast of KwaZulu-Natal and 550 km further north in Inhambane on the Mozambique coast [56].

#### 3.2.5. Geckos

On the other hand, the Gekkonidae, also a very speciose reptile family, only second to Scincidae, are more often introduced (Table 1). Three gecko species—Cape dwarf gecko *Lygodactylus capensis*, Bibron’s gecko *Pachydactylus bibronii* and marbled leaf-toed gecko *Afrogecko porphyreus*—were unintentionally introduced (translocated) from one part of Africa to another. The Farquhar half-toed gecko *Hemidactylus mercatorius* was introduced from Madagascar to Reunion probably through stowaway. Only one species, the Indo-Pacific slender gecko *Hemiphylodactylus typus*, was introduced (through stowaway) from another region of the world (SE Asia) to islands around Madagascar.

### 3.3. Species Which Failed to Develop Viable Populations

There is a global trend for the importing and keeping of alien pets, especially reptiles, which may result in the subsequent release/escape of some of these animals into the wild. Many amphibian and reptile pet species kept in Africa could have escaped or were intentionally released into the wild. Since they have failed to develop viable populations, and subsequently disappeared or became extinct, they have passed unrecorded. However, there has been an exponential increase in imports from an increasing number of originating countries [12,57].

In a few countries, introduction events have been more closely monitored; e.g., in South Africa, a total of 275 species of alien reptiles and 14 amphibians have been reported so far. Only two snake species, the olive snake *Elaiophis inornatus* and brown house snake *Baedon capensis*, were recently introduced to Africa. It is, however, not confirmed whether they have developed viable populations in the areas of introduction (South Africa). Most introduced reptiles and amphibians were recorded in four provinces: KwaZulu-Natal (n = 112 spp.), the Western Cape (104 species), the Eastern Cape (65) and Gauteng (number of species not available). The most popular pet species in this country are snakes (167) and lizards (77) [12].

With the advent of a greater level of awareness, we are only now discovering and noticing translocated reptiles. The proper documentation of this information is important in order to avoid unsubstantiated conclusions. With increases in human and trade mobility, it is becoming even more important to keep the records, so that it will be known when a species has been introduced, and subsequently becomes established or invasive.

**Table 1:** Familiar affinities of alien amphibians and reptiles recorded in sub-Saharan Africa. The number of amphibian species in the world is according to [58], and that of reptiles according to [59].

Taxa	All Species in the World	Alien Species in Sub-Saharan Africa				Total No. of the Alien spp.	% Out of All spp. in the World
		Established		Not Established			
	n	n	%	n	%	n	%
**AMPHIBIA**	8973	10	23.3	11	31.4	21	0.23
**Caudata**	836	0	0.0	4	11.4	4	0.48
Salamandridae	147	0	0.0	3	8.6	3	2.04
Ambystomatidae	32	0	0.0	1	2.9	1	3.13
**Anura**	7917	10	23.3	7	20.0	17	0.21
Pipidae	41	1	2.3	0	0.0	1	2.44
Bufonidae	666	4	9.3	0	0.0	4	0.60
Rhacophoridae	462	1	2.3	0	0.0	1	0.22
Hyperoliidae	236	2	4.7	0	0.0	2	0.85
Ptychadenidae	63	1	2.3	0	0.0	1	1.59
Pyxicephalidae	91	1	2.3	0	0.0	1	1.10
Ceratophryidae	12	0	0.0	1	2.9	1	8.33
Microhylidae	764	0	0.0	1	2.9	1	0.13
Hylidae	762	0	0.0	2	5.7	2	0.26
Dendrobatidae	213	0	0.0	3	8.6	3	1.41
**REPTILIA**	12,502	33	76.7	24	68.6	57	0.46
**Testudines**	366	5	11.6	4	11.4	9	2.46
Emyidae	58	1	2.3	1	2.9	2	3.45
Testudinidae	47	3	7.0	1	2.9	4	8.51
Chelydridae	5	0	0.0	1	2.9	1	20.00
Trionychidae	36	1	2.3	0	0.0	1	2.78
Pelomedusidae	27	0	0.0	1	2.9	1	3.70
**Sauria**	7905	26	60.5	7	20.0	33	0.42
Agamidae	604	2	4.7	1	2.9	3	0.50
Chamaeleonidae	234	1	2.3	4	11.4	5	2.14
Iguanidae	45	0	0.0	1	2.9	1	2.22
Gekkonidae	1713	23	53.5	0	0.0	23	1.34
Scincidae	1793	0	0.0	1	2.9	1	0.06
**Serpentes**	4203	2	4.7	12	34.3	14	0.33
Pythonidae	40	0	0.0	1	2.9	1	2.50
Boidae	67	0	0.0	1	2.9	1	1.49
Colubridae	2167	1	2.3	7	20.0	8	0.37
Lamprophiidae	93	0	0.0	2	5.7	2	2.15
Viperidae	406	0	0.0	1	2.9	1	0.25
Typhlopidae	425	1	2.3	0	0.0	1	0.24
**Crocodylia**	27	0	0.0	1	2.9	1	3.70
Crocodylidae	17	0	0.0	1	2.9	1	5.88
Total	21,475	43	100.0	35	100.0	78	0.36

## 4. Places of Introduction

Most alien amphibian and reptile species were introduced to South Africa (n = 57), mostly to the Cape Town, Durban and Witwatersrand areas, and small islands around Madagascar (Malagasy Region), such as Reunion (n = 19), Mauritius (n = 17), Rodrigues (n = 8) and Comoros (n = 5). Five alien species have been recorded so far in Kenya and 4 species in Tanzania. In other countries only 1–2 species have been recorded to date. It is important to point out that this distribution of recorded introduced species reflects both the importance of these areas for the introduction of alien species, as well as the level of reporting alien species. Certainly, in many African countries, especially those with large sea ports, most introductions/releases pass undetected or unrecorded. This may, however, not apply to established alien species. It can be, therefore, concluded that most established alien species exist on the small islands in the Malagasy Region, especially in Reunion and Mauritius. This remains true also in regard to alien birds [11] and mammals [12].

## 5. Places of Origin of Introduced Species

**Figure 1:**
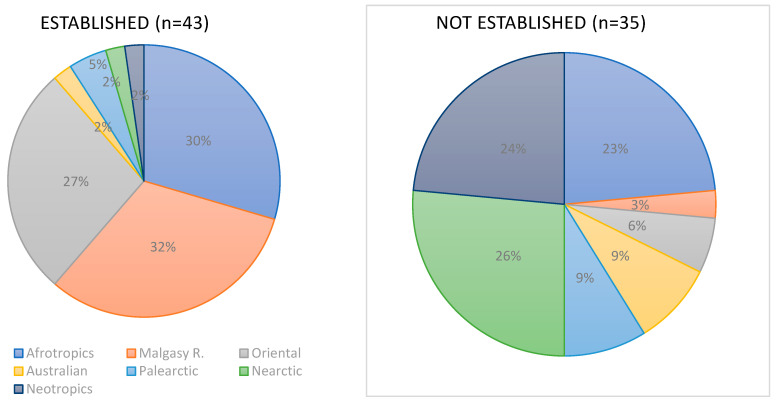
Original zoogeographical regions of amphibian and reptile species introduced to sub-Saharan Africa.

Most species (62%) introduced to sub-Saharan Africa and established there originated from other regions of Africa, including the Malagasy Region, and from the Oriental Region (27%). Only 11% of these species originated from other regions of the world (Figure 1). The proportions are quite different in the case of species introduced to sub-Saharan Africa, but not established (Figure 1). Most of them originated from the New World (i.e., Nearctic and Neotropic Regions, together 50%) and from other parts of Africa (23%).

## 6. Timing of Introduction

In Africa, most introductions of small reptile and amphibian species are non-intentional, except for species used as pets (e.g., *Phelsuma grandis*). Consequently, alien reptiles are often detected several years after their introduction [43,60], so their exact origins remain uncertain [32].

**Figure 2:**
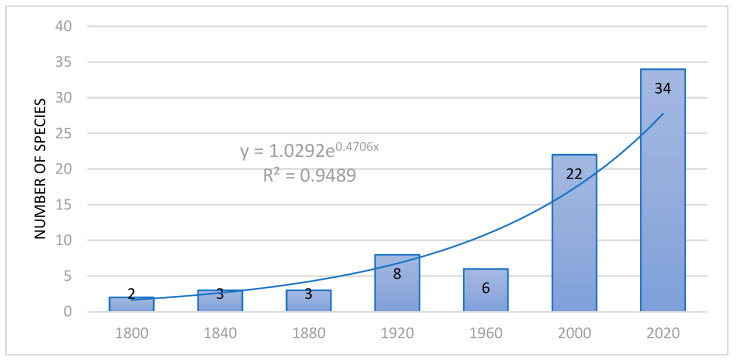
Years of introductions of amphibians and reptiles to sub-Saharan Africa (*p* < 0.01).

**Figure 3:**
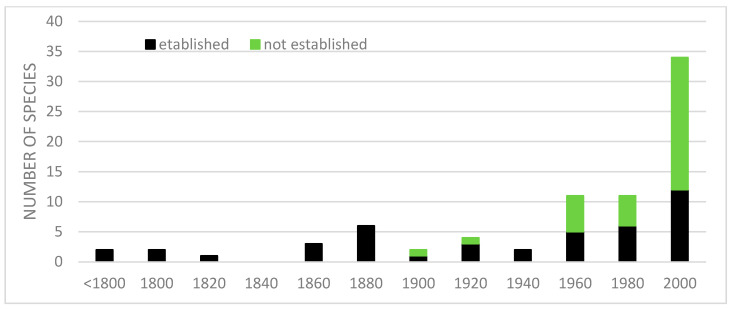
Years of successful (n = 43) and unsuccessful (n = 35) introductions of amphibians and reptiles to sub-Saharan Africa.

The first introduction of alien herp species to sub-Saharan Africa, i.e., the common four-clawed gecko *Gehyra mutilata* and Mascarene green frog *Ptychadena mascareniensis*, took place in 18th century. Toward the end of 19th century, four other species were introduced and in the two last decades of that century, five other species. This unusual increase was probably due to a scheme of intentional introduction carried out by colonial authorities. Similarly, in the 20th century, most introductions were made in the last two decades, when an exponential growth of introduction begun, lasting until the present day (Figure 2). This growth has been caused by an increase in international trade and herp pet industry, especially in South Africa. The growth is, however, quite different for the established and non-established species (Figure 3). While the number of established species grew slowly over more than 200 years, the number of non-established species grew more rapidly over less than 100 years (Figure 3).

## 7. Pathways of Introduction

### 7.1. Stowaways

It is clear that most reptile and amphibian individuals were introduced unintentionally (Table 2). Geckos, tree frogs and reed frogs have particular properties suitable for being transferred. They are capable of crossing large distances on vehicles unintentionally. Historical records suggest that very often this has been the case in southern Africa. There is an anecdotal record of introductions occurring, albeit at a low frequency, over a long period. However, most of these accidental introductions involved single individuals (no propagule pressure), and invasion had no chance to occur.

Examples of some southern African frog species that have been moved out of their range include: (a) the foam-nest tree frog which was associated with bananas and other fruits; (b) the red toad which was regularly transported in luggage. The foam-nest tree frog is capable of surviving long trips, but would not be likely to start invasive populations unless propagule pressure to a suitable breeding site was increased. Introductions of these frogs, and other species of tree frogs and reed frogs, will likely increase as trade increases. Adult red toads are common in peri-urban areas and have a propensity to climb inside shoes and suitcases and are subsequently being moved large distances by humans to new areas. Toads (and presumably other anurans tolerant of desiccation or high salinity) are capable of surviving ocean crossings inside containers. The Asian common toad *Duttaphrynus melanostictus* arrived in South Africa in this way, as it arrived in other countries [61,62].

The introduction of *Lygodactylus capensis* to Cape Town is thought to have originated with the establishment of a population in a nursery. Hitch-hiking and stowaways as adults and eggs are likely to be the pathway of these invasions [41].

### 7.2. Pet Trade

The capture of reptile and amphibian species for the pet trade is regarded as the second largest threat to these species [63]. Mainland Africa supports more than 571 snake species [59]. Between 2013 and 2017, a total of 2269 wild snakes represented by 42 species from 15 African countries were advertised for sale on the internet. The main hubs for trade are located in Egypt, Madagascar, Tanzania, and Togo [64,65].

Today it is illegal to import alien reptile and amphibians to South Africa, but since the 1980s, shipments of amphibians have been sent there for the pet trade. Over 300 reptile and amphibian species have been imported to South Africa as pets [12]. These animals were quickly disseminated throughout the country, in both the pet and scientific trade, resulting in occasional introductions into the wild. Fortunately, almost all these released animals did not establish viable populations. Although it is illegal to import alien reptiles and amphibians, it does not necessarily mean that the pet trade no longer takes place in the country.

Pet trade creates potentially more opportunities than stowaways for both intentional and unintentional introductions, as usually more species are offered in pet trade. However, successful introduction depends to a large extend on the propagule pressure, and this may be higher in the case of stowaway introductions. Stowaways (dispersal from sea ports) appear to be a more successful way of introduction in small oceanic islands, whereas the pet trade can be more effective in inland introductions, especially in deep interior areas (landlocked countries) and in more developed and more populated countries, such as South Africa, due to a higher frequency of the pet trade.

### 7.3. Leading-Edge (Jump) Dispersal

Some species of African amphibians are prone to being moved large distances, e.g., the guttural toad and painted reed frog (Table 2). In South Africa, invasive amphibians use artificial water bodies, such as impoundments or garden ponds, as a resource that facilitates reproduction and dispersal through a stepping-stone movement.

In the case of the painted reed frog both human-mediated jump dispersal and diffusion-based leading-edge dispersal play a role in the range expansion. This species’ jump dispersal into new localities is caused mainly by accidental translocation in nursery plants and aquatic plants. The painted reed frog is, however, also able to travel long distances over land and this may contribute to the leading-edge dispersal between water bodies.

The African clawed frog has expanded its natural distribution in southern Africa through colonization of farm dams, irrigation channels and other artificial water bodies also through a leading-edge dispersal, e.g., [66].

### 7.4. Cultivation Dispersal

The axolotl *Ambystoma mexicanum* (Appendix A), originating from Mexico, was bred successfully in some laboratories and then traded throughout the world as a pet since about 1864 [67]. This can be called cultivation dispersal, as almost all traded animals originate from six animals imported to Paris in 1864 [67].

## 8. Factors Determining Introduction Success

### 8.1. Behavioral and Morphological Traits

Species that are easy to breed and handle or are large, colorful or patterned are preferred as pets. The most common alien snake in captivity in South Africa is therefore the corn snake. Of course it occurs also in the wild in this country in some urban areas, although it is still not established. This is probably because it has conspicuous coloration, which attracts the attention of pet breeders. Similarly conspicuous coloration of geckos from the genus *Phelsuma* attracts the attention of herp breeders. As a consequence, there is a higher likelihood of unintentional or intentional release of these animals into the wild.

### 8.2. Propagule Pressure

Propagule pressure (the number of introduced individuals) is an important factor determining the establishment of alien reptile and amphibian species. It can be the main reason why most escapees from terraria cannot develop viable populations [12]. For instance, up to 2011, only one alien reptile species recorded in South Africa, the Brahminy blind snake, has established a viable population [23].

### 8.3. Climate and Habitat Overlap

If climate (rainfall and temperature averages, minima and maxima) and habitat are unsuitable, a species will not establish a viable population, regardless of propagule pressure. For instance, the climate in western and central Africa is suitable for establishment of the green iguana, but the climate in southern Africa is not suitable for establishment of this species [12]. The painted reed frog exhibits a high desiccation resistance and plasticity of thermal tolerance, which may enable the establishment of a viable population in drier and thermally less stable habitats, such as those in the Western Cape in South Africa [68].

### 8.4. Presence of Potentially Competing Species

On islands, invasions take place 110 times more frequently and with a higher probability of viable population establishment than on the mainland [18]. According to [69,70], reptile communities with a high number of species are on islands more resistant to invasion by exotic reptile species than communities with fewer reptile species. Predation and competition probably set their barriers on the distribution, colonization and abundance for alien species. For example, the Cape Verde Islands are relatively poor in reptile species diversity [71,72], so the introduction of two house gecko species (*Hemidactylus angulatus* and *H. mabouia* [72] caused a decline in the indigenous Cape Verde Leaf-toed Gecko *Hemidactylus bouvieri* [73]. On the other hand, out of 571 alien animal species recorded in mainland South Africa, only one herp species, the Brahminy blind snake, was regarded as established by 2015 [23].

## 9. Impacts of Introduced Amphibians and Reptiles on Local Fauna

In sub-Saharan Africa, introduced amphibians and reptiles may cause ecological damage to native biodiversity through competition, predation, hybridization and transmission of parasites and diseases. However, the impacts are usually not appropriately evaluated, because these evaluations are not based on abundance, distribution, and population trend studies. The type of ecological impact is not specified for some alien species. No information is given on whether this represents a research gap or the impact was investigated, but not evidenced. Introduction events in some African countries may be underreported due to insufficient monitoring.

### 9.1. Competition

There are several evidenced cases of the competition between alien and indigenous herp species. However, in sub-Saharan Africa, no information is available on indirect effects such as changes in food web structure or impacts on ecosystem functioning. In the Western Cape, South Africa, the introduced guttural toad competes for habitat and food resources with the endangered indigenous western leopard toad (*Sclerophrys pantherina*) [74]. In South Africa and Reunion, the common slider may compete with native turtles for food and basking sites, while the common house gecko is known to displace other smaller gecko species [75]. In Mauritius, the Indian wolf snake competes with indigenous snakes [32]. The green tree lizard is known to compete with indigenous gecko species [38]. The cane toad competes for food with native anuran species [2].

The conservation status of the endangered Cape platanna rests, in part, on the threats produced by sympatric populations of the common platanna which competes with Cape platanna [76]. The common agama in the Comoro Islands (introduced there in 1990) affects indigenous and endemic day geckos (*Phelsuma* spp.), Comoro Island skink (*Trachylepsis comorensis*) and Cuvier’s Madagascar swift (*Oplurus cuvieri comorensis*). Although the species was introduced to the Township of Moroni only, at present it occurs in different parts of the island reducing the population size and home range of the above-mentioned reptile species. It can cause cascading effects in native ecosystems by disrupting trophic interactions and sharing ecological resources with native species.

In Mauritius and Reunion, introduced day geckos encroach on the habitats of the blue-tailed day gecko *Phelsuma cepediana*, lowland forest day gecko *Phelsuma guimbeaui*, ornate day gecko *Phelsuma ornata*, and upland forest day gecko *Phelsuma rosagularis* [77]. The Arabian leaf-toed gecko *Hemidactylus homoeolepis* introduced to Socotra competes with the indigenous *Hemidactylus* spp. [78]. The Madagascar giant day gecko released from a nursery in Reunion in 1994, and Mauritius in the 1990s, is a possible threat to indigenous lizards [75]. The common house gecko *Hemidactylus frenatus* introduced in Mauritius caused a decline of endemic *Phelsuma* day geckos and local extinction (through food competition) of the endemic *Nactus* night geckos [75].

### 9.2. Predation

Predation of alien species on threatened indigenous species is suspected in some alien herp species, although there are only few recorded cases of such predation. The guttural toad, introduced to Mauritius and Reunion, preys on some threatened native invertebrates [2]. In Mauritius, the Indian wolf snake preys on native reptiles [12]. The cane toad preys upon some endangered species [2,74], while the African clawed frog preys on the indigenous Cape platanna in the Western Cape [74]. *Phelsuma* day geckos, especially *P. grandis*, introduced to Mauritius and Reunion were observed not only competing, but also preying upon endemic *Phelsuma* geckos living on these islands, such as *P. borbonica*, *P. cepediana*, *P. guimbeaui*, *P. ornata* and *P. rosagularis* [75,77].

### 9.3. Hybridization

Released alien pythons may hybridize with the indigenous rock python *Python sebae* [12]. The African clawed frog hybridizes with the indigenous Cape platanna in the Western Cape, South Africa [79]. This genetic pollution is quite common and may threaten the Cape platanna [80]. No other cases of hybridization have been recorded so far, but this phenomenon may pass undetected.

### 9.4. Transmission of New Diseases and Parasites

Alien species may transmit new diseases and new parasites against which indigenous animals are not immune. However, only a few cases have been hitherto recorded. The African clawed frog may transmit to the indigenous Cape platanna a particularly vicious chytridiomycosis caused by *Batrachochytrium dendobatidis*. Alien turtles, especially the common slider and the soft-shelled Chinese terrapin, increase the risk of trematode and protozoan (e.g., transmission *Haemogregarina stepanova*) infestation to the native species [81,82,83,84].

## 10. Comparison with Other Regions

Globally, a total of 78 amphibian and 198 reptile species have become established outside their native ranges, i.e., about 1% of amphibian and 1.9% of reptile species. Regions most affected by alien amphibians are the UK, France, and Hawaii (7–8 established species), whereas those most affected by reptiles (>10 species) are: Japan, Florida, California, the Balearic Islands, Hawaii, and the Bahamas; 9–10 spp.: Spain and Mauritius, Reunion; 7–8 species: Madagascar and Texas. The most widespread introductions include the following amphibian species: *Rhinella marina*, *Eleutherodactylus johnstonei*, *Xenopus laevis*, *Eleutherodactylus planirostris*, and *Osteopilus septentrionalis*. Meanwhile, *Ramphotyphlops braminus*, *Trachemys scripta*, *Hemidactylus frenatus*, *Hemidactylus mabouia*, *Hemidactylus turcicus*, *Anolis sagrei*, *Podarcis siculus*, *Tarentola mauritanica*, *Gehyra mutilata*, and *Hemidactylus garnotii* are among the most widely introduced reptile species [18,19,85].

In Europe, a total of 29 amphibian and 48 reptile species were introduced and became established. The countries with the highest number are the UK (20 amphibian and 88 reptile species introduced, with 14 and 23 established; the London Area, c. 6400 km^2^, is the center of these introductions) and Spain (26 amphibians and 49 reptiles, including 17 and 20 established species) [85]. In Central Europe, 16 alien reptile species have been recorded [85], with *Anolis carolinensis*, *Lampropeltis getula*, *Pantherophis guttatus*, *Telescopus fallax* and *Vipera ammodytes* posing the highest establishment risk.

For North America, 100 introduced and subsequently established species were listed, including two salamander, 18 anuran, 12 turtle, 60 lizard, seven snake and one crocodilian species [9]. Florida is the state with the highest number of alien species. A total of 180 alien herp species have been recorded there, including 4 Urodela, 13 Anura, 27 turtle, 4 crocodilian, 92 lizard and 40 snake species [86,87].

For comparison, a total of 57 reptile and 21 amphibian species have been recorded in sub-Saharan Africa, although only 19 and 10 species respectively have become established. These numbers are comparable to Spain, are much lower than in Florida, and much lower still than in Europe and North America. This is possibly because the Afrotropical Region is saturated with herps which can potentially compete and prey on alien species, preventing their successful establishment.

Madagascar, the Mascarenes and other small islands in the Malagasy Region have the highest number of introduced herp species in sub-Saharan Africa. However, the numbers are still much lower than those recorded for instance in the Greater Caribbean, which has 130 introduced species, i.e., 25 amphibians and 105 reptiles [88], including five amphibian and 21 reptile species in the largest island, Cuba [89].

## 11. Management Implications

Intentional introductions (release) should be prohibited by law before a risk assessment has been carried out on a species-by-species basis, as a risk assessment for every species imported to any country is not operational. In South Africa, it is now illegal to import alien amphibians and reptiles. A risk assessment protocol should be implemented for categorizing species as permissible or prohibited for import and trade in each country. For highly dangerous species, a ban on import, trade and possession should be considered.

Preventing introductions is a key issue of nature conservation. It is widely considered to be the most cost-effective way to combat invasions and needs to be promptly organized at the national, continental and international scales. The prevention requires, however, a certain level of knowledge about frequency and ways of species trade, and the identification of taxa in which invasion risks and potential impacts on native ecosystems are the highest.

Invasive alien species impacts on small islands are especially acute and are exacerbated through agricultural intensification, urban development, over-exploitation, and climate change [90,91,92,93]. Eradication is an effective way to combat invasive species on islands, but it can be effective only at early stages of invasion [94].

On small islands (archipelagos), such as the Mascarenes, Comoros, and Seychelles, stronger collaboration is advocated among island countries and territories with similar socio-ecological environments facing the same invasion alien species.

The introduction of an invasive species’ natural predator into their new range has been used to control it, but sometimes with negative effects. Given the risks associated with biological control methods, it should be considered as a last solution to the problem [95]. Risk-averse management tools include risk maps. These should elaborated, taking into account climatic and habitat suitability, entry points, expansion limitations, and ability to reproduce in the new ecosystem. Such maps may help in locating potential invasive species hotspots and aid in prevention of invasive species establishment. Adult invasive frogs should be eliminated by trapping or hand capture and their tadpoles may be destroyed by draining water bodies or chemical treatment. This can be done, however, only if the species is the only amphibian species living in the area, and it is not applicable everywhere but should be decided on a case-by-case basis, and not as a general solution.

Fencing may also be used to limit frog movements away from infested habitats [96]. In order to restrict access to aircraft and ships, reptiles and amphibians should be inspected in outbound cargo using specially trained dogs and other methods. Developing and potential methods include the use of oral toxicants, repellents, reproductive inhibition, and barriers [4]. South Africa has introduced legislation focusing on preventing the influx of invasive species into the country and on managing invasive species which are already in the country [12]. In general, the following steps are recommended in combating alien amphibians and reptiles: (1) prevention, (2) early detection and rapid response, (3) eradication, (4) control and management, and (5) restoration [90,91].

Eradication of invasive species from small oceanic islands around Africa is of the highest conservation priority [97,98,99]. With a goal of collating these efforts, the Database of Islands and Invasive Species Eradications [98] has been established to hold records of the location, target species, year and outcome of invasive species on particular islands. The database holds records for more than 2000 eradication attempts as of December 2019 [98].

## 12. Conclusions

Although in recent decades, introduction of alien reptiles and amphibians accelerated on a global scale, mainland Africa is fortunate to have escaped their invasions. Such conditions exist despite the fact that most African countries do not use any national legislation or programs to control invasions. Even in South Africa, where many introductions took place, there are only a few established alien species (e.g., the Indian wolf snake) (Appendix A). This is probably because of high diversity of indigenous herp species in the mainland, which outcompete the introduced species, and also high predation rate by native mammals, birds and reptiles. Contrary to this, the small islands around sub-Saharan Africa, especially those around Madagascar, have been invaded by many species which subsequently became well established and some of them even invasive. Most of these invasive species are representative of the Gekkonidae family. There is an urgent need to closely monitor and control the invasions of reptiles and amphibians, especially on islands, as the process of their invasion is increasing.

## Figures and Tables

**Table 2:** Pathways of introduction of alien amphibians and reptiles in sub-Saharan Africa. Explanations: est.—established, not est.—not established.

Way of Introduction	Amphibians		Reptiles		Total	
	Est.	Not Est.	Est.	Not Est.	Est.	Not Est.
Pet release	7	5	9	12	16	17
Unintentional translocation	1	2	1	6	2	8
Intentional introduction	0	0	3	0	3	0
Stowaway	0	3	20	2	20	5
Biological control	2	0	0	0	2	0
Unknown	0	1	0	4	0	5
Total	10	11	33	24	43	35

## Data Availability

No new data were created or analyzed in this study. Data sharing is not applicable to this article.

## Appendix A

**Table A1:** Amphibian and reptile species introduced to sub-Saharan Africa.

Scientific Name	Common Name	Family	Natural Range	Inv.	Place and Date of Introduction	Source
AMPHIBIANS						
*Cynops pyrrhogaster*	Japanese fire-bellied newt	Salamandridae	Japan	3	South Africa: KwaZulu-Natal, Cape Town, 2016	[13]
*Triturus cristatus*	Crested newt	Salamandridae	Europe	3	South Africa, 2010s	[13]
*Notophthalmus viridescens*	Eastern newt	Salamandridae	E North America	3	South Africa: W Cape, 2010s	[13]
*Ambystoma mexicanum*	Axolotl	Ambystomatidae	Mexico	4	South Africa: Bloemfontein, 1980s, now extinct	[100]
*Xenopus laevis*	African clawed frog	Pipidae	Africa	1	South Africa: W Cape, 1908s	[79]
*Sclerophrys gutturalis*	African common toad	Bufonidae	Southern Africa	1	Reunion (1927), Mauritius (1922), South Africa: Cape Town (2000), Kenya: Nairobi (2025)	[74,101,102]
*Rhinella marina*	Cane toad	Bufonidae	Neotropics	1	Mauritius, 1922	[2]
*Schismaderma carens*	African red toad	Bufonidae	S and E Africa	2	SA: Cape Town stowaway, 2013	[13]
*Duttaphrynus melanostictus*	Asian common toad	Bufonidae	SE Asia	2	South Africa: W Cape: Tokai, Bellville, 2012; Tomasina (Madagascar), 2015	[13,103]
*Chiromantis xerampelina*	Gray foam-nest tree frog	Rhacophoridae	S and E Africa	2	South Africa: Stellenbosch, Porterville, Victoria West, 2012	[13]
*Hyperolius marmoratus*	Painted reed frog	Hyperoliidae	Mozambique, Malawi, SA	2	South Africa: W Cape: Villiersdorp (1997) and Cape Town (2004)	[50]
*Hyperolius tuberilinguis*	Tinker reed frog	Hyperoliidae	S and E Africa	2	South Africa: Cape Town, Bloemfontein, 2009	[13]
*Ptychadena mascareniensis*	Mascarene grass frog	Ptychadenidae	Madagascar	2	Mauritius, Reunion, before 1790; Seychelles, 2010s;	[104,105]
*Pyxicephalus adspersus*	African bull frog	Pyxicephalidae	S Africa	2	South Africa: Cape Peninsula: Muizenberg Mountains, 1980s	[79]
*Ceratophrys ornata*	Argentine horned frog	Ceratophryidae	Argentina, Uruguay	3	South Africa, c. 2008/2009	[13]
*Dyscophus antongilii*	Madagascar tomato frog	Microhylidae	Madagascar	3	South Africa: Transvaal Snake Park, 1990s	[13]
*Dendrobates leucomelas*	Yellow-banded poison dart frog	Dendrobatidae	N South America	3	South Africa: Johannesburg: Montecasino Bird Gardens, 2010s	[13]
*Dendrobates auratus*	Green-and-black poison dart frog	Dendrobatidae	Central America	3	South Africa: Johannesburg: Montecasino Bird Gardens; uShaka; Two Oceans, 2010s	[13]
*Dendrobates tinctorius*	Dyeing poison dart frog	Dendrobatidae	N South America	3	South Africa: Johannesburg: Montecasino Bird Gardens, 2010s	[13]
*Litoria albolabris*	Wandolleck’s white-lipped tree frog	Hylidae	New Guinea	3	South Africa, 2010s	[13]
*Litoria caerulea*	Australian green tree frog	Hylidae	Australia	3	South Africa, 2010s	[13]
REPTILES						
*Crocodylus niloticus*	Nile crocodile	Crocodylidae	Africa	3	South Africa, 1960s	[106]
*Macrochyles temmincki*	Alligator snapper turtle	Chelydridae	USA	4	South Africa: George, 2000s, now extinct	[12]
*Palea steindachneri*	Wattle-necked softshell turtle	Trionychidae	China, Laos Vietnam	2	Mauritius, 1980s Reunion, SA	[107,108]
*Trachemys scripta*	Common slider	Emydidae	E USA	1?	South Africa: Pretoria/Johannesburg and Durban areas; Reunion, Mauritius, Seychelles, 1990s; Kenya: Nairobi	[109,110]
*Emys orbicularis*	European pond turtle	Emydidae	Europe	3	South Africa (E Cape), 1990s?	[106]
*Pelusios subniger*	East African black mud turtle	Pelomedusidae	E Africa	4	Mauritius, Seychelles, 1970s, now extinct	[111]
*Geochelone platynota*	Burmese star tortoise	Testudinidae	SE Asia	4	Kenya, now extinct	[106]
*Astrochelys radiata*	Radiated tortoise	Testudinidae	S Madagascar	2	Mauritius (Rodrigues, Round Island), Reunion, 1980s; Rodrigues, 2006	[15,17]
*Geochelone pardalis*	Leopard tortoise	Testudinidae	Africa	2	South Africa, 1960s?	[106]
*Aldabrachelys gigantea*	Aldabra giant tortoise	Testudinidae	Seychelles: Aldabra atol	2	Rodrigues: François Leguat Reserve Rodrigues, 2006	[107]
*Indotyphlops braminus*	Brahminy blind snake	Typhlopidae	SE Asia	1	Tanzania; Durban, SA: Cape Town, 1920s; Reunion, beg. 19th cen.?; Mauritius; Seychelles; Annobon, 2003	[72,106,112,113,114,115]
*Python molurus bivittatus*	Burmese python	Pythonidae	SE Asia	4	Cape Town, 2000s, now extinct	[12]
*Boa constrictor*	Boa constrictor	Boidae	South America	4	South Africa, 2000s, now extinct	[106,109]
*Lycodon aulicus*	Indian wolf snake	Colubridae	India	1	Reunion, beg. 19th cen.?; Mauritius (1879)	[116,117]
*Lampropeltis californiae*	Californian king snake	Colubridae	North America	4	South Africa, 2000s	[118]
*Lampropeltis triangullum*	Sinaloan king snake	Colubridae	North America	4	South Africa, now extinct, 2000s	[118]
*Lampropeltis alterna*	Gray-banded kingsnake	Colubridae	Mexico	4	South Africa: Strand, 2000s, now extinct	[12]
*Pantherophis guttatus*	Red corn snake	Colubridae	SE USA	4	South Africa: Durban, Johannesburg, Cape Town, 2000s, now extinct	[12]
*Elaphe obsoleta spiloides*	Gray rat snake	Colubridae	E and C USA	4	South Africa: Durban, 2000s, now extinct	[12]
*Elaphe obsoleta quadrivittata*	Yellow rat snake	Colubridae	USA	4	South Africa: Cape Town, 2000s, now extinct	[12]
*Pituophis m. melanoleucus*	Northern pine snake	Colubridae	SE USA	4	South Africa: Durban, 2000s, now extinct	[12]
*Elaiophis inornatus*	Olive snake	Lamprophidae	Africa	3	South Africa, 2000s	[12]
*Baedon capensis*	Brown house snake	Lamprophiidae	Southern Africa	3	South Africa, 2000s	[12]
*Crotalus atrox*	Western diamond-backed rattlesnake	Viperidae	SW USA, N Mexico	4	South Africa: Gauteng, 2000s, now extinct	[12]
*Furcifer pardalis*	Panther chameleon	Chamaeleonidae	Madagascar	2	Reunion, before 1830	[119]
*Bradypodion pumilum*	Cape dwarf chameleon	Chamaeleonidae	Africa	3	Namibia, 1990s: Swakopmund and Walvis Bay, also Lüderitz, Windhoek, now extinct	[25,120]
*Calumma parsonii*	Green giant chameleon	Chamaeleonidae	N Madagascar	3	Mauritius, 1960s	[20]
*Chamaeleo quilensis*	Flap-necked chameleon	Chamaeleonidae	South Africa: KZN	3	South Africa: Free State, before 1978	[109,121]
*Bradypodion* spp.	Dwarf chameleon	Chamaeleonidae	South Africa: KZN	3	South Africa: Free State, 1939	[106,121]
*Cryptoblepharus boutoni*	Bouton’s snake-eyed skink	Scincidae	Eastern coast of Africa	3	South Africa: KZN, 1990s	[106]
*Iguana iguana*	Green iguana	Iguanidae	Neotropics	4	South Africa: Gauteng, 2000s, now extinct	[12]
*Agama agama*	Common agama	Agamidae	E Africa	1	Reunion, mid. 19th cen.; Comoro Isl., 1997; Madagascar, 2004	[36,122,123]
*Pogona vitticeps*	Bearded dragon	Agamidae	Australia	4	South Africa, 2000s, now extinct	[116]
*Calotes versicolor*	Green tree lizard	Agamidae	Indonesia	1	Mascarenes (Rodrigues, Mauritius, Reunion), 1865; Seychelles; Keyna: Nairobi, end of 20th cen., 1920	[38,124] iNaturalist
*Gehyra mutilata*	Common four-clawed gecko	Gekkonidae	SE Asia	1?	Reunion, 18th cen.?; Mauritius, Rodrigues, Seychelles	[14,125]
*Phelsuma laticauda*	Gold dust day gecko	Gekkonidae	N Madagascar	1	Reunion, 1975; Mauritius, Mayotte, Comoros, Seychelles	[45,126]
*Phelsuma astriata*	Seychelles day gecko	Gekkonidae	Seychelles	1	Reunion, before 2003	[47,127,128]
*Phelsuma grandis*	Madagascar giant day gecko	Gekkonidae	N Madagascar	1	Reunion, 1994; Mauritius, 1990s	[75,129]
*Phelsuma lineata*	Lined day gecko	Gekkonidae	Madagascar	1	Reunion, 1940	[47]
*Phelsuma dubia*	Dull day Gecko	Gekkonidae	Madagascar, Seychelles	2	Comoros Isl., coast of E Africa; 2000s	[45]
*Phelsuma borbonica agalegae*	Agalega day gecko	Gekkonidae	Agalega Isl. (Mauritius)	2	Reunion, 2000s	[45]
*Phlesuma cepediana*	Blue-tailed day gecko	Gekkonidae	Mauritius	1	Rodrigues, Madagascar, 2000s	[45]
*Tarentola mauritanica*	Moorish wall gecko	Gekkonidae	Mediterranean Region	1	S Western Sahara, 1970s,	[130]
*Lepidodactylus lugubris*	Common mourning gecko	Gekkonidae	Indo-Pacific	1	Seychelles, Rodrigues, 1960s	[14]
*Lygodactylus capensis*	Cape dwarf gecko	Gekkonidae	S, E Africa	2	Central Africa, 1956; Tanzania: Pemba; South Africa: Free State, before 1978	[13,121]
*Chodrdactylus turneri*	Turner’s thick-toed gecko	Gekkonidae	South Africa: KZN	2	Free State, before 1978	[99,131,132]
*Ebenavia boettgeri*	West Madagascan clawless gecko	Gekkonidae	E Madagascar	1?	Mauritius, 1960s	[14]
*Hemidactylus mercatorius*	Farquhar half-toed gecko	Gekkonidae	Madagascar, Seychelles	1	Cape Verde (Santo Antao, Sao Vincente), Comoro Islands, Europa Is., Reunion, Mauritius, Rodrigues, Mayotte, 1960?	[14]
*Hemidactylus* *mabouia*	Tropical house gecko	Gekkonidae	W and C Africa	1	Principe, São Tome, 1884; Bazaruto; Free State, before 1978; Simon’s Town, 1962; Gordon’s Bay, 1976; East London, Port Elizabeth,	[30,41,132,133,134]
*Hemidactylus* *longicephalus*	Long-head half-toed gecko	Gekkonidae	C, W Africa	1	Principe, São Tome, 1892	[135]
*Hemidactylus flaviviridis*	Northern house gecko	Gekkonidae	SE Asia	1	Socotra, end of 19th cen.?; only known from Hadiboh town and outskirts	[136]
*Hemidactylus robustus*	Heyden’s gecko	Gekkonidae	SW Asia, Horn of Africa	1	Socotra, end of 19th cen.	[137]
*Hemidactylus homoeolepis*	Arabian leaf-toed gecko	Gekkonidae	S Arabian Peninsula	1	Socotra, 1990s	[78]
*Hemidactylus frenatus*	Common house gecko	Gekkonidae	India, Sri Lanka	1	Reunion (19th cen.), Mauritius, Rodrigues, Seychelles, Comoros, Madagascar, South Africa, Somalia	[14,112]
*Hemidactylus parvimaculatus*	Small spotted house gecko	Gekkonidae	S India, Sri Lanka	2	Mauritius, Reunion, Rodrigues, Comoro, end of 19th century; Seychelles	[14,138]
*Hemiphylodacty-lus typus*	Indo-Pacific slender gecko	Gekkonidae	SE Asia	2	Reunion, 1960s?; Mauritius, Rodrigues, Comoro Isl. (Moheli)	[14]
*Afrogecko porphyreus*	Marbled leaf-toed gecko	Gekkonidae	South Africa	2	South Africa: Cape Town, Port Elizabeth, 2010s	[41]

Inv. = invasiveness: (1) established, developed viable population; (2) not established although developed small isolated viable population; (3) released, but still has not developed viable population; (4) released into the wild, it could even have established/developed viable population, but now extinct.
